# How to select IgG subclasses in developing anti-tumor therapeutic antibodies

**DOI:** 10.1186/s13045-020-00876-4

**Published:** 2020-05-05

**Authors:** Jifeng Yu, Yongping Song, Wenzhi Tian

**Affiliations:** 1 Department of Hematology, The First Affiliated Hospital of Zhengzhou University, Zhengzhou, 450052 China; 2 Academy of Medical and Pharmaceutical Sciences of Zhengzhou University, Zhengzhou, 450052 China; 3 ImmuneOnco Biopharmaceuticals (Shanghai) Co., Ltd., Shanghai, 201203 China

**Keywords:** Therapeutic antibody, IgG subclass, Fcγ receptor, ADCC, ADCP

## Abstract

The intact antibody of human immunoglobulin (IgG) is composed of the fragment for antigen binding (Fab) and the crystallizable fragment (Fc) for binding of Fcγ receptors. Among the four subclasses of human IgG (IgG1, IgG2, IgG3, IgG4), which differ in their constant regions, particularly in their hinges and CH2 domains, IgG1 has the highest FcγR-binding affinity, followed by IgG3, IgG2, and IgG4. As a result, different subclasses have different effector functions such as antibody-dependent cell-mediated cytotoxicity (ADCC) and antibody-dependent cellular phagocytosis (ADCP). Fcγ receptors include six subtypes (FcγRI, FcγRIIA, FcγRIIB, FcγRIIC, FcγRIIIA, FcγRIIIB) which differ in cellular distribution, binding affinity to Fc, and the resulting biological activity. Therefore, when developing anti-tumor therapeutic antibodies, including single-targeted antibodies, bi-specific antibodies (BsAbs), and antibody-drug conjugates (ADCs), many factors, such as target biology, cellular distribution of the targets, the environments of particular tumor types, as well as the proposed mechanism of action (MOA), must be taken into consideration. This review outlines fundamental strategies that are required to select IgG subclasses in developing anti-tumor therapeutic antibodies.

## Background

Due to its high specificity and superior pharmacokinetics, the antibody drug has attracted great attention across the pharmaceutical industry since the late 1990s, especially following the inaugural approval of rituximab, the first anti-tumor antibody drug, in 1997. Anti-tumor antibody drugs exert therapeutic efficacy by several different, but not mutually exclusive, mechanisms [1], including (1) Fc-mediated effector functions (ADCC, ADCP, CDC); (2) blocking tumor growth signals; (3) inhibiting angiogenesis; (4) triggering apoptotic pathways in tumor cells; (5) activating immune cells. Among the FDA-approved 22 anti-tumor antibodies (Table 1) [2–24], almost half (10) of them engage in anti-tumor activity via Fc-mediated effector functions. The other half engaged via other mechanisms such as apoptosis induction or tumor growth signal blockage antibodies targeting immune checkpoints (such as PD-(L)1 antibodies) usually act by neutralizing inhibitory signals [10], while CTLA-4 antibody ipilimumab activates immune function by depleting regulatory T cells (Tregs) from tumor microenvironment (TME) [11, 12].

**Table 1:** FDA approved tumor-therapeutic antibodies [2–24]

Targeted drugs	IgG subclass	Target	Indications	Mechanism of action	Refs
Rituximab (Rituxan)	IgG1	CD20	NHL	ADCC/CDC	[2]
Trastuzumab (Herceptin)	IgG1	Her2	Breast cancer	Growth signal blocking; ADCC	[3, 4]
Cetuximab (Erbitux)	IgG1	EGFR	mCRC	Growth signal blocking; ADCC	[5]
Bevacizumab	IgG1	VEGF	Solid tumors	Angiogenesis inhibition	[6]
Panitumumab (Vectibix)	IgG2	EGFR	mCRC	Growth signal blocking	[7]
Ofatumumab (Arzerra)	IgG1	CD20	CLL	ADCC/CDC	[8]
Alemtuzumab (Campath)	IgG1	CD52	CLL	ADCC/CDC	[9]
Denosumab (Xgeva)	IgG1	RANKL	Bone tumor	Growth signal blocking	[10]
Ipilimumab (Yervoy)	IgG1	CTLA-4	Solid tumors	Depleting Treg cells	[11, 12]
Pertuzumab (Perjeta)	IgG1	Her2	Breast cancer	Growth signal blocking	[13]
Obinutuzumab (Gazyva)	IgG1	CD20	CLL	ADCC; apoptosis induction	[14]
Ramucirumab (Cyramza)	IgG1	VEGFR2	Solid tumors	Angiogenesis inhibition	[15]
Pembrolizumab (Keytruda)	IgG4	PD-1	Solid tumors	Neutralizing inhibitory signal in T cells	[16]
Nivolumab (Opdivo)	IgG4	PD-1	Solid tumors	Neutralizing inhibitory signal in T cells	[17]
Dinutuximab (Unituxin)	IgG1	GD2	Neuroblastoma	ADCC/CDC	[18]
Daratumumab (Darzalex)	IgG1	CD38	Multiple myeloma	ADCC/CDC; apoptosis induction	[19]
Elotuzumab (Empliciti)	IgG1	SLAMF7	Multiple myeloma	ADCC,direct activation of NK cells	[20]
Atezolizumab (Tecentriq)	IgG1	PD-L1	Solid tumors	Neutralizing inhibitory signal in T cells	[21]
Avelumab (Bavencio)	IgG1	PD-L1	Solid tumors	Neutralizing inhibitory signal in T cells; ADCC	[22]
Durvalumab (Imfinzi)	IgG1	PD-L1	Solid tumors	Neutralizing inhibitory signal in T cells	[21]
Mogamulizumab (Poteligeo)	IgG1	CCR4	CTCL	ADCC	[23]
Cemiplimab (Libtayo)	IgG4	PD-1	Solid tumors	Neutralizing inhibitory signal in T cells	[24]

IgG has four subclasses, named IgG1, IgG2, IgG3, and IgG4 [25]. Although all the subclasses have more than 90% identity on the amino acid level, each subclass however has a unique profile with respect to the length of hinge region, the number of inter-chain disulfide bonds, and Fc-effector functions. Differences in the profile of IgG subclasses are summarized in Table 2 [26]. Among the four IgG subclasses, IgG3 demonstrates the highest affinity binding to most FcγRs, but is not selected routinely as a therapeutic format due to its long hinge region and polymorphic nature, both of which increase the risk of stability and immunogenicity [28], thus IgG3 will not be discussed further in this review. Of the remaining subclasses, IgG1 demonstrates the highest affinity for all FcγRs and is a potent activator of ADCC and ADCP. IgG4 only has high affinity for FcγRI but weak affinities for all other receptors, and is a poor inducer of Fc-mediated effector functions. IgG2 has high affinity for the H131 form of FcγRIIA, but no measurable or weak affinity for FcγRI and all other FcγRs (Table 2).

**Table 2:** Properties of human IgG subclasses and relative binding affinity with major FcγRs [26, 27]

	IgG1	IgG2	IgG3	IgG4
Molecular mass (kD)	146	146	170	146
Amino acids in hinge region	15	12	62	12
Inter-heavy chain disulfide bonds	2	4	11	2
Complement activation (C1q binding)	++	+	+++	—
FcγRI	650	—	610	340
FcγRIIa-H131	52	4.5	8.9	1.7
FcγRIIa-R131	35	1	9.1	2.1
FcγRIIb	1.2	0.2	1.7	2
FcγRIIIa-V158	20	0.7	98	2.5
FcγRIIIa-F158	11.7	0.3	77	2

*(x10^5^M^_1^) [27]

Human Fcγ receptor family includes six members (FcγRI, FcγRIIA, FcγRIIB, FcγRIIC, FcγRIIIA, and FcγRIIIB) which differ in cellular distribution, binding affinity to Fc, and the resulting biological activity. The most functionally relevant FcγRs are FcγRI, FcγRIIA, FcγRIIB, and FcγRIIIA, thus FcγRIIC and FcγRIIIB will not be discussed in this review. Except for FcγRI, which is an activating receptor with high affinity for IgG and can be activated by monomeric IgG, all other FcγRs can only be functionally engaged by the Fc region of IgG when complexed with a specific antigen. FcγRIIB is the only inhibitory FcγR and is expressed on many immune cells including B cells, monocytes, macrophages, DCs, and mast cells (Table 3). When engaged with antigen-bound IgG, FcγRIIB transduces an inhibitory signal through the intracellular immunoreceptor tyrosine-based inhibitory motif (ITIM), resulting in inhibition of ADCC and ADCP [30]. The best characterized activating FcγRs are FcγRIIA and FcγRIIIA. FcγRIIA is primarily expressed on monocytes and macrophages, while FcγRIIIA is dominantly expressed on NK cells. Both the activating FcγRs have two allelic variants (Table 2), with FcγRIIA-H131 having an overall higher affinity for Fc than FcγRIIA-R131, and FcγRIIIA-V158 having a higher affinity for Fc than FcγRIIIA-F158 [27, 29].

**Table 3:** Cellular distribution of the major human FcγRs [29]

FcγR	Cellular distribution
FcγRI	Monocytes, macrophages, activated neutrophils, DCs
FcγRIIa	Monocytes, macrophages, neutrophils, DCs
FcγRIIb	B cells, monocytes, macrophages, DCs, mast cells
FcγRIIIa	NK cells, monocytes, macrophages

When developing cancer therapeutic antibodies, an appropriate subclass of human IgG must be selected to couple with the proposed MOAs. In this review, strategies for selection of IgG subclass based on the cellular distribution and biological natures of the targets in the development of single-targeted antibodies, bispecific antibodies (BsAbs), as well as ADC drugs will be discussed.

## Selection of IgG subclasses for single-targeted antibodies

Antibodies against single targets for cancer therapy have been promising, especially for those with appropriately selected IgG subclass. Although there are not stringent criteria for the selection of IgG subclass for a single-targeted antibody, cellular distribution and the biological function of the targets should be considered when selecting the subclass.

## Selection of IgG subclasses for tumor cell targets

Up to 12 targets expressed on tumor cells have been clinically validated (Table 1). Except for EGFR, for which both IgG1 and IgG2 were respectively selected for the formats of cetuximab and panitumumab, all other targets were paired with IgG1. Although Her2 and EGFR belong to the tyrosine kinase receptor family for which a targeted antibody may exert anti-tumor activity by blocking the growth signal, the ADCC and/or ADCP induced by the IgG1 antibodies (e.g., cetuximab and trastuzumab) against RTKs also play substantial therapeutic roles. Thus, when developing therapeutic antibodies against targets expressed on tumor cells, IgG1 should be primarily considered, for it has the best binding affinity for activating FcγRs and can elicit strong ADCC and/or ADCP activity against tumor cells. Additionally, when ADCP is activated, the effector cells (macrophages or dendritic cells) may present the processed tumor antigen to T cells, thus further eliciting a long-lasting tumor-specific adaptive immunity [31].

Some tumor cell targets are ligands for immune checkpoints (ICPs), such as PD-L1 and CD47, which are the interacting partner for PD-1 and SIRPα, respectively. PD-1 and its ligand PD-L1 perform a vital role in tumor progression and survival by escaping tumor neutralizing immune surveillance. PDL-1 is expressed in tumor cells and antigen-presenting cells, and the engagement of PDL1 with PD-1 of T cell creates T cell dysfunction, exhaustion, neutralization, and interleukin-10 (IL-10) production in a tumor mass [32]. Therefore, blocking PD-1 or PD-L1 recognition process can block the inhibition signaling, consequently reactivate the effector T cells to kill tumor cells [33]. Although simply blocking PD-L1 could induce significant therapeutic efficacy, retention of the Fc-effector function in the IgG1 format would further improve the therapeutic efficacy. It was reported that Avelumab, an IgG1 anti-PD-L1 inhibitor, triggers NK cell-mediated cytotoxicity and cytokine production against triple-negative breast cancer cells [22].

Optimal induction of ADCC was dependent on the abundance of target expression on tumor cells, for which the minimal requirement per cell is 10^5^-10^6^. Cells with target density less than 10^5^ are less likely to induce and thus will not be damaged by ADCC. Normal tissues usually express much lower level of the targets compared to tumor tissues [34].

## Selection of IgG subclasses for targets expressed in immune cells

Selection of IgG subclasses for immune cell targets is more complicated because some targets act to regulate immune function by transducing inhibitory signals to T cells. These targets are immunoinhibitory receptors which are collectively referred to as immune checkpoints (ICP) (such as CTLA4, PD-1, TIM-3, LAG-3). Meanwhile, others are targets for activating immune cells (4-1BB, CD40, OX40, CD27, GITR, etc.), usually called immunostimulatory receptors. Among the best characterized immune checkpoints, PD-1, TIM-3, and LAG3 are mainly expressed in CD8 T lymphocytes, while CTLA-4 is primarily expressed in Treg cells in TME. As such, different strategies of IgG format should be carefully considered when developing therapeutic antibodies against different ICP. For example, IgG4 (but not IgG1) should be the primary antibody format for PD-1 antibody (all the currently approved PD-1 antibodies are in the IgG4 subclass format.) However, since IgG4 only has a high affinity to FcγRI but weak affinity to all other FcγRs and thus will not have a detectable Fc-mediated effector function meaning the CD8 T lymphocytes will not be eliminated accordingly, IgG1 should be selected for those ICPs mainly expressed in Tregs or other immunosuppressive cells, which can be depleted by the antibodies via the mechanism of ADCC/ADCP. Ipilimumab targeting CTLA-4 is such an antibody that exerts therapeutic efficacy mainly via ADCC/ADCP eliminating Tregs from TME [11, 12] so that more room can be made for effector T cells that can be activated by PD-1 antibodies. There is one IgG2 antibody, tremelimumab, targeting CTLA-4 that has been under extensive clinical trials since 2006 [35]. Since IgG2 only has high affinity to FcγRIIa-H131 that is mainly expressed in macrophages that will not be fully activated in the absence of CD47-SIRPα blockers, tremelimumab would not be able to eliminate Tregs from TME, and thus would not be effective in cancer therapy. Point of fact, tremelimumab has unfortunately already failed six late-stage clinical trials thus far (Table 4) [36] https://www.pfizer.com/news/press-release/press-release-detail/pfizer_announces_discontinuation_of_phase_iii_clinical_trial_for_patients_with_advanced_melanoma., https://www.astrazeneca.com/media-centre/press-releases/2016/astrazeneca-reports-top-line-result-of-tremelimumab-monotherapy-trial-in-mesothelioma-29022016.html#!, https://www.astrazeneca.com/media-centre/press-releases/2018/astrazeneca-reports-results-from-the-arctic-trial-in-third-line-non-small-cell-lung-cancer-24042018.html, https://www.astrazeneca.com/media-centre/press-releases/2018/astrazeneca-provides-update-on-the-phase-iii-mystic-trial-of-imfinzi-and-tremelimumab-in-stage-iv-non-small-cell-lung-cancer16112018.html, http://www.pharmabiz.com/NewsDetails.aspx?aid = 112740&sid = 2, https://www.marketscreener.com/ASTRAZENECA-4000930/news/AstraZeneca-Update-on-the-Phase-III-NEPTUNE-trial-29093768/. However, another study demonstrated that ipilimumab and tremelimumab increase the infiltration of intratumoral CD4+ and CD8+ cells without significantly changing or depleting FOXP3+ cells within the tumor microenvironment. It suggests that anti-CTLA-4 immunotherapy does not deplete FOXP3+ cells in human tumors, but their efficacy could be enhanced by modifying the Fc portions of the monoclonal antibodies to enhance Fc-mediated depletion of intratumoral regulatory T cells [37].

**Table 4:** Summary for failure of clinical trials for tremelimumab [36]

Indications	Therapy	Stage	Code	Refs
Advanced melanoma	Monotherapy	Phase IIb	A3671009	https://www.astrazeneca.com/media-centre/press-releases/2018/astrazeneca-reports-results-from-the-arctic-trial-in-third-line-non-small-cell-lung-cancer-24042018.html
Mesothelioma	Monotherapy	Phase IIb	DETERMINE	https://www.astrazeneca.com/media-centre/press-releases/2018/astrazeneca-provides-update-on-the-phase-iii-mystic-trial-of-imfinzi-and-tremelimumab-in-stage-iv-non-small-cell-lung-cancer16112018.html
Previously treated NSCLC	Durvalumab plus tremelimumab	Phase III	ARCTIC	http://www.pharmabiz.com/NewsDetails.aspx?aid = 112740&sid = 2
Previously untreated NSCLC	Durvalumab plus tremelimumab	Phase III	MYSTIC	https://www.marketscreener.com/ASTRAZENECA-4000930/news/AstraZeneca-Update-on-the-Phase-III-NEPTUNE-trial-29093768/
HNSCC	Durvalumab plus tremelimumab	Phase III	EAGLE	[37]
Previously untreated NSCLC with high TMB	Durvalumab plus tremelimumab	Phase III	NEPTUNE	[38]

Most of the immunostimulatory receptors belong to the tumor necrosis factor receptor (TNFR) superfamily (such as 4-1BB, CD40, OX40, CD27, GITR), which upon engagement by agonistic antibodies, can stimulate immunity. In contrast to antibodies for immune checkpoint receptors (such as PD-1) for which pure target blocking can activate T cells, activation of a TNFR-superfamily member by antibody requires hyper cross-linking of the antibodies with FcγRs. For example, anti-CD40 mAb requires inhibitory FcγRIIB-mediated crosslinking for agonistic effect [38–40]. It has been documented that the anti-CD40 human IgG1 antibody produces potent antitumor activity in tumor models with the mFcγR−/−*hFCGR2B* genotype, depleting (by N297A mutation) or increasing (by S267E mutation) the binding affinity to hFcγRIIB, respectively abrogating or enhancing anti-tumor activity [38]. This suggests FcγRIIB is absolutely required for antibodies targeting immunostimulatory receptors of TNFR superfamily members [41–44]. Additionally, the hinge region in the CH1 domain is also important and required for antibody agonistic function. The more rigid the hinges of a given IgG, the more stable the clustering of the immunostimulatory receptors on cell membranes and thus the greater the anti-tumor efficacy [45]. Replacement of the hinge region with that of IgG3 completely eliminated the anti-tumor activity of the anti-CD40 antibody, although both the CD40 and the FcγR binding affinity were retained. Even for the engineered IgG1-Fc with enhanced binding affinity to FcγRIIB [46], the original potent anti-tumor activity was completely lost. However, when combining the rigid hinge region with an engineered Fc domain stronger for FcγRIIB binding, anti-tumor activity significantly improved [45], indicating human CH1-hinge regions, selected for rigidity, and Fc domains engineered for FcγRIIB engagement can synergize to enhance the immunostimulatory and anti-tumor activities of antibodies targeting TNFR superfamily members.

Safety is also a major concern for agonistic Abs since one antibody, urelumab, targeting 4-1BB has been stopped for clinical trials following the occurrence of two hepatotoxicity-related deaths [47]. Another 4-1BB antibody, utomilumab [48], showed better safety profiles but is less potent relative to urelumab, with no encouraging efficacy data has been observed thus far as a monotherapy. Analysis on these two Abs indicated that urelumab is a fully human IgG4 with a hinge mutation (S228P) to improve stability [49, 50] and is a more potent agonist since it can co-stimulate T cells in the absence of FcγR [51, 52], while utomilumab, a fully human IgG2 [48], requires FcγRIIB-expressing cells for its agonistic activity, and thus is a weak agonistic Ab. Accumulated clinical data was not promising for either antibody [53]. Efforts have been made to mitigate the liver toxicity and improve the efficacy of 4-1BB antibodies [52, 54] by engineering the Fc portion of the antibody to eliminate binding to the activating FcγRs, including FcγRI, FcγRIIA, and FcγRIIIA, while retaining binding to the inhibitory Fc receptor FcγRIIB [52]. Alternatively, antibodies targeting different epitopes away from the ligand-binding sites may have better efficacy as well as safety profiles if they are IgG4, which have better binding affinity to FcγRIIB than IgG2, which only binds to FcγRIIA [54].

Collectively, the IgG1 subclass should be preferentially considered when developing antibody drugs targeting tumor antigens, especially when the Fc-mediated effector function is the main mode of action for tumor therapy. Selection of IgG subclass format for targets in immune cells should be done with careful consideration to the nature of the targets. If ICPs are expressed on effector immune cells, then IgG4 should be preferentially considered. If expressed on Tregs or other immunosuppressive cells such as M2 macrophages or myeloid-derived suppressive cells (MDSC), then IgG1 should be selected. For targets with immunostimulatory function, especially for those of the TNFR superfamily members, IgG should be engineered to have enhanced FcγRIIB engagement besides epitope screening.

## Selection of IgG subclass format in bispecific antibody development

Due to the limitations of the treatment response as well as therapeutic efficacy for single target-specific antibodies in addressing cancer indications, bispecific antibody (BsAb) drug development has emerged as an attractive focus of biopharmaceutical companies globally. Although the concept of dual-targeted therapy is promising, translation of the concept into therapeutic products is challenging in several aspects: (1) CMC is complex due to mismatch of the heavy and light chains from respective monoclonal antibodies; (2) target pairing is scientifically challenging due to the general lack of detailed study on the synergy of the selected targets; (3) structural format selection needs to be extensively tested in vitro as well as in vivo before final format can be determined, especially since the nature and the cellular distribution of the two targets addressed by the developing BsAb are complex.

**Fig. 1:**
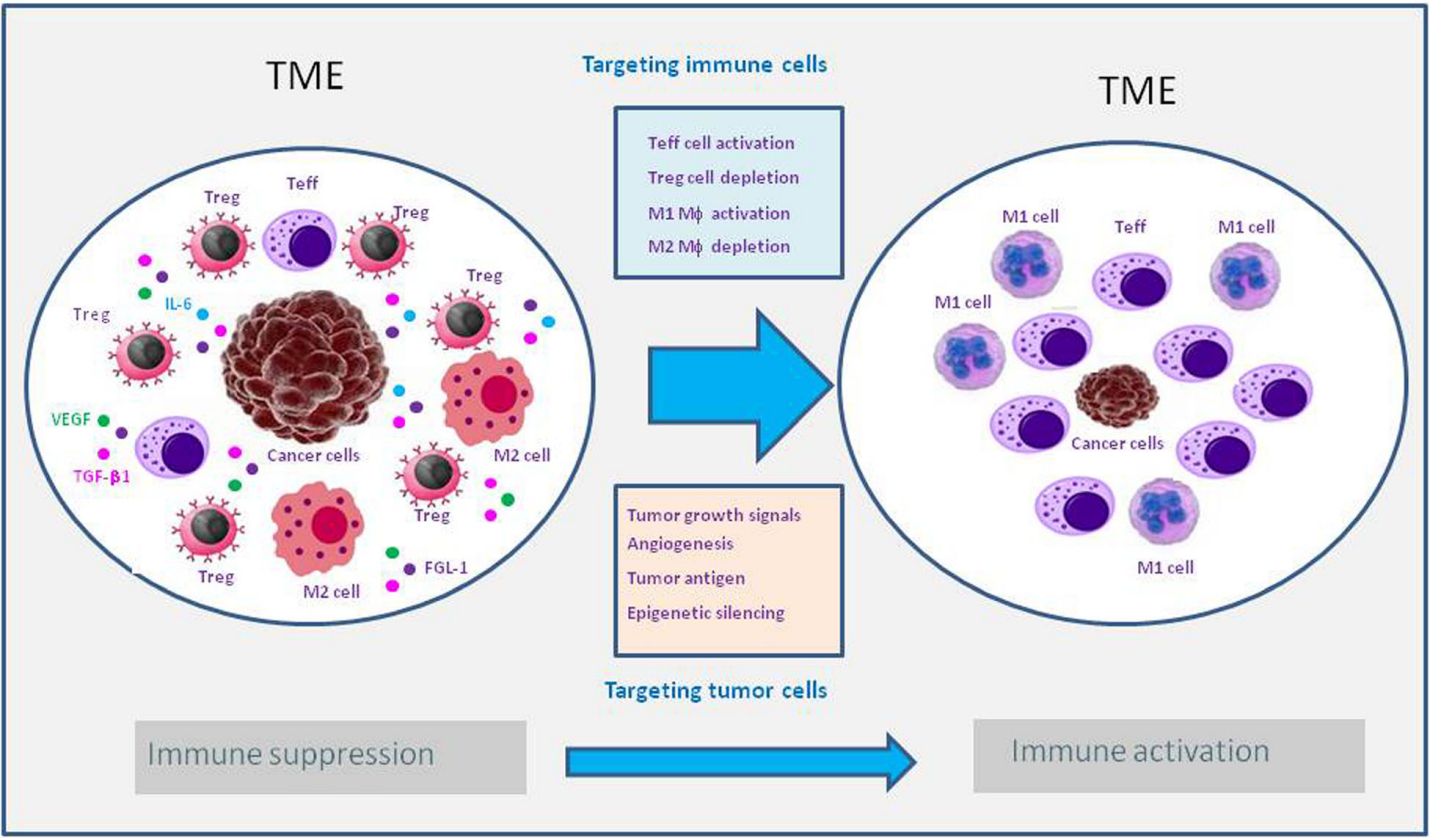
Depiction of tumor microenvironment. Tumor microenvironment (TME) is specially built up by tumor cells creating an ideal environment for tumor cell growth. TME is full of immunosuppressive cells (such as Tregs, M2 macrophages) and soluble factors (such as VEGF, TGF-β, FGL-1) secreted by tumor cells that play roles in promoting angiogenesis and tumor growth, in induction of Tregs and M2 macrophages. Tumor cells can also initiate epigenetic silencing pathways preventing immune cells from infiltration into TME. The best rationale for developing BsAb would be to simultaneously inhibit tumor growth and activate long-lasting immunity against tumors

Additionally, the design of effective bispecific therapeutic molecules should take into full consideration the characteristics of the tumor microenvironment (TME) where a lot of immune suppressive cells reside along with tumor cells that are smart and can always find a way to evade immune cell attack (Fig. 1). For instance, tumor cells can upregulate the ligands for ICPs such as PD-L1 [55, 56] and FGL-1 [57], upregulate epigenetic silencing genes such as enhancer of zeste homolog 2 (EZH2) [58], and/or secrete a large amount of soluble immune suppressive factors such as TGF-β and VEGF [59, 60]. Therefore, rationales of target as well as IgG format selection in BsAb development in general should be built upon the following factors: (1) biological synergy of the targets; (2) cellular distributions; and (3) Fc-mediated effector functions (such as ADCC/ADCP). Arbitrary construction of a dual-target molecule without thoroughly understanding the back-end mechanisms will have less likelihood of clinical success.

## Selection of IgG subclasses for BsAb against two tumor cell targets

If both targets are membrane-bound receptors (especially RTK targets) expressed by tumor cells, the IgG1 subclass should be selected. If the corresponding interacting partner of one or both tumor targets is an ICP (such as PD1, SIRPα), IgG1 format will undoubtedly have better efficacy since this format of BsAb could (1) block tumor growth signals; (2) activate innate immune cells via Fc-FcγR interaction-mediated ADCC/ADCP; (3) activate adaptive immune cells (if one of the interacting partners is ICP such as PD-1).

**Fig. 2:**
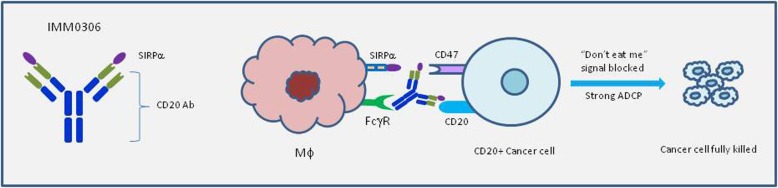
Working model of CD47xCD20 Bispecific Molecule (IMM0306). IMM0306 targeting CD47xCD20 (Left) is composed of CD20 antibody and the first domain of SIRPα which is connected to the N-terminal of the heavy chain of the antibody. The Fc domain was engineered to have enhanced ADCC/ADCP activity. When administrated, IMM0306 will block the “don't eat me” signal by binding to CD47 and blocking CD47-SIRPα interaction between cancer cells and macrophages, in parallel, it will also strongly activate innate immune cells via Fc-FcγRIIA/FcγRIIIA interaction leading to fully killing of the cancer cells

Taking our CD47xCD20 bispecific molecule (IMM0306) as an example (unpublished data), both CD47 and CD20 are highly expressed in tumor cells (in this case, tumor cell is a B cell), as such, IgG1 format was selected to (1) block the “don't eat me” signal resulting from CD47-SIRPα interaction; and (2) activate innate immune cells via Fc-FcγR interaction (Fig. 2) (unpublished data). In vivo administration of the bispecific molecule at low dose (1.5 mg/kg) in Daudi xenograft model in SCID mice resulted in complete eradication of the established tumors in 8 of 8 mice, while treatment with rituximab at the dose of 5 mg/kg led to tumor eradication only in 3 of 8 mice (data not shown). The depletion of macrophages dampened the efficacy of IMM0306, but it was still significantly better than rituximab at the same calculated dose (data not shown), suggesting NK cell-mediated ADCC might account for the difference.

## Selection of IgG subclasses for BsAb against two immune cell targets

If both targets are expressed in immune cells, selecting a subclass becomes much more complicated. To do so, it is necessary to distinguish the biological activity of the targets as well as the types of immune cells.

If both targets are immune checkpoints and are expressed on effector (CD4/CD8) T lymphocytes (e.g., PD-1xTIM-3, PD-1xLAG-3), or on two different immunoactivating cells (such as effector T cells, NK cells (PD-1xNKG2A), and M1 macrophages (PD-1xSIRPα)), the Fc-mediated effector function should be absolutely avoided, thus IgG2 or IgG4 should be selected. Alternately, the BsAb molecule should exclude the Fc domain so that the activating immune cells would not be eliminated by ADCC/ADCP.

If both targets are expressed on Treg cells (such as CTLA-4xGITR), or one on a Treg cell (CTLA-4) and the other on a MDSC cell (such as CSF1R), or both ICP targets on tumor cells (e.g., PD-L1xCD47), then IgG1 should be selected so that the immunosuppressive cells (Treg, MDSC) or tumor cells over-expressing ICPs (e.g., PD-L1xCD47) can be eliminated via IgG1-mediated ADCC/ADCP.

Pairing of the two targets for BsAb development is also very important and needs to undergo thorough scientific scrutiny before selection. If the two targets were miss-matched, then the clinical efficacy would not be reached as originally anticipated, no matter what structural format of the BsAb is selected. One example of this case is the development of BsAbs targeting PD-1xCTLA-4. Since PD-1 antibody (mostly IgG4) plays its role by blocking the PD-1 signal, and CTLA-4 antibody (IgG1) plays its role mainly by eliminating Treg cells via ADCC/ADCP activity, it is thus unreasonable to develop a BsAb targeting these ICPs, because no matter what IgG subclass (IgG1 or IgG4) is selected, it is impossible to achieve the expected effect. If IgG1 is selected, both Treg cells (highly expressed CTLA4) and PD-1 cells (mainly CD8 T cells) will be eliminated in parallel. If IgG4 is selected, only PD-1 target will be blocked, resulting in T cell activation because simply blocking CTLA-4 signal has no therapeutic effect [11, 12] as exampled by tremelimumab [61, 62]. Proposed IgG subclass for selected target pairs is listed in Table 5.

**Table 5:** Proposed IgG subclass for selected target pairs

Targets	Proposed IgG
PD-1xTIM-3	IgG4
PD-1xLAG-3	IgG4
PD-1x4-1BB	IgG4, Fc engineering for FcγRIIB engagement
PD-1xCD40	IgG4, Fc engineering for FcγRIIB engagement
PD-1xOX40	IgG4, Fc engineering for FcγRIIB engagement
PD-1xNKG2A	IgG4
PD-1xSIRPa	IgG4
PD-L1xCTLA-4	IgG1, Fc engineering for FcγRIIA/FcγRIIIA engagement
CTLA-4xGITR	IgG1, Fc engineering for FcγRIIA/FcγRIIIA engagement
CTLA-4xCSF1R	IgG1, Fc engineering for FcγRIIA/FcγRIIIA engagement
PD-L1xCD47	IgG1, Fc engineering for FcγRIIA/FcγRIIIA engagement
CD3xTarget2	IgG4, KIH format

## Selection of IgG subclasses for BsAbs respectively against one tumor cell targets and one immune cell targets

There is a situation that the two targets are expressed by a tumor cell and an immune cell, respectively. In this case, CD3-based bispecific antibodies are most common, such as CD3xCD19 [63], CD3xCD20 [64], CD3xBCMA [65], CD3-HAC [66]. Because of the expression of CD3 in T cells, if the structural format is IgG-like, then IgG1 must not be selected. Otherwise, T cells will be eliminated. Instead, IgG2 or IgG4 should be selected and the BsAb was assembled by KIH (knob into hole) platform technology [67].

In recent years, bispecific antibodies have been the popular focus for both preclinical and clinical studies [68–70]. There are many bispecific antibodies in the clinical development stages. As of March 2020, there are a total of 55 registered active clinical trials with different bispecific antibodies from the www.ClinicalTrials.gov website. Although only two BsAb drugs https://www.drugs.com/newdrugs/fda-approves-blincyto-blinatumomab-precursor-b-cell-acute-lymphoblastic-leukemia-4115.html, https://www.drugs.com/newdrugs/fda-approves-hemlibra-emicizumab-kxwh-hemophilia-inhibitors-4639.html have been approved by the FDA thus far, it is anticipated that as least five more BsAb drugs could be approved in the coming years.

## Selection of IgG subclass format in ADC drug development

Antibody-drug conjugates (ADCs) are complex molecules composed of antibodies conjugated with cytotoxic payloads via chemical linkers. The well-described mechanism of action for ADC drugs includes [68, 71, 72] (1) binding of the mAb to the target in tumor cells; (2) internalizing the ADC into the cells through receptor-mediated endocytosis; (3) releasing the cytotoxic payload from the internalized ADC to kill the target cells [73]. The critical requirements for ADC drugs include unique expression of the targets on tumor cells but no or minimal expression in normal tissues, stable linkers that can only be cut inside of the target cells, and targets able to internalize upon antibody binding. While all the relevant information has been well documented, the selection of IgG subclass format for ADC development was less discussed previously.

Although the primary efficacy of ADC drugs is mediated by the toxic payloads delivered by antibodies, the Fc-mediated effector functions may also contribute to the therapy, which is impacted by the expression and cellular distribution of the targets. Among the currently approved five ADC drugs [74] (Table 6), two employed IgG4 antibodies which are lack of effector functions, while all other three employed IgG1, of which brentuximab vedotin has been reported to induce ADCP in vivo and contribute to the potent anti-tumor efficacy for this ADC [75, 76]. However, other studies have identified Fc receptor engagement by ADC may cause side effects. For example, T-DM1 has been demonstrated to be internalized by megakaryocytes in vivo via FcγRIIA engagement and resulted in the development of thrombocytopenia [77, 78]. Thus, selection of IgG subclass for ADCs should make into consideration of the type of tumors as well as the expression of the targets. For liquid tumors and the targets are restricted only in tumor cells, IgG1 should be selected; otherwise, IgG2 or IgG4 should be primarily considered.

**Table 6:** FDA approved antibody-drug-conjugates (ADCs) [74]

ADC drugs	Target	Indications	Antibody conjugation	Approval date
Adcetris® (brentuximab vedotin)	CD30	Relapsed HL and sALCL	Chimeric IgG1—cysteine	2011
Kadcyla® (trastuzumab emtansine)	Her2	Breast cancer	Humanized IgG1—lysine	2013
Besponsa® (inotuzumab ozogamicin)	CD22	r/r B-ALL	Humanized IgG4—lysine	2017
Polivy® (polatuzumab vedotin)	CD79b	r/r DLBCL	Humanized IgG1—cysteine	2019
Enhertu® (fam-trastuzumab deruxtecan-nxki)	Her2	Breast cancer	Humanized IgG1—cysteine	2019

## Strategies for manipulating Fc-mediated functions

It has been well confirmed that antibodies with enhanced ADCC activity against some particular targets showed significantly better therapeutic efficacy, while antibodies with increased binding affinity for FcγRIIB showed stronger immune-activating activity if the targets are TNFR superfamily members. Thus, engineering the Fc domain to manipulate the Fc-mediated function has been a hot focus across the pharmaceutical industry.

Several antibodies lacking fucose have been developed either by engineering the Fc domain or by production in cell lines lacking the fucosyltransferase gene. For example, the CD20 antibody obinutuzumab and the CCR4 antibody mogamulizumab were respectively produced in CHO cells lacking the fucosyltransferase gene [79, 80] or CHO cells lacking both the fucosyltransferase and the fucosyltransporter genes [81]. The removal of the fucose from the Fc portion increased binding affinity with the 158F allele of the FcγRIIIA receptor. Other examples are margetuximab [82] for Her2, and Tafasitamab [83] for CD19, both were developed to enhance ADCC activity by engineering the Fc domain. Clinical trials for the two antibodies all met the primary ends and both were recently submitted to the FDA for marketing approval recently. Margetuximab was reported to have enhanced binding affinity to FcγRIIIA-158F, but a decreased affinity for FcγRIIB, an inhibitory receptor, which allows it to bind more tightly to effector cells and increase ADCC [84]. When compared with Herceptin, the overall survival (OS) for margetuximab-treated breast cancer patients expressing FcγRIIIA-158F was extended by 4.3 months (23.7 months vs 19.4 months) [85].

## Conclusions

In summary, the Fc-FcγR interaction of a given IgG Fc plays the same important role as the Fab-antigen interaction for anti-tumor antibodies. Thus when designing anti-tumor therapeutic antibodies, extensive consideration should be given to the biological characteristics of the targets, the corresponding cells expressing the targets, and the mechanism of actions proposed for the therapeutic antibodies. If ADCC/ADCP is a must, then IgG1 should be selected, and in most of the cases, the Fc portion should be engineered to increase the binding affinity for the activating FcγRs (FcγRIIA, FcγRIIIA) and/or remove the binding affinity for the inhibitive FcγR (FcγRIIB). For the immunostimulatory receptors, especially the tumor necrosis factor receptor superfamily receptors (such as CD40, 4-1BB, OX40), improved Fc-FcγRIIB engagement of the IgG (IgG1 or IgG4) with relatively weak agonistic activity would be the ideal choice for balancing the efficacy and toxicity of the antibodies. In the case of the bispecific antibodies, consideration should be centered on the synergistic effects of the corresponding single-target antibodies and the mechanism of action of the BsAbs, besides the biological characteristics and the cellular distribution of the two targets. Finally, proper pairing of the two targets for BsAb development is of upmost importance. Blindly pairing two targets and arbitrary construction of a dual-target molecule without thoroughly understanding the back-end mechanisms will undoubtedly have less likelihood of clinical success.

## Data Availability

This is not applicable for this review.

## Abbreviations

ADCC: Antibody-dependent cell cytotoxicity
ADCP: Antibody-dependent cell phagocytosis
BsAb: Bispecific antibody
TME: Tumor microenvironment
